# Nissen Fundoplication: 70 years of a simple and brilliant idea in the surgical treatment of gastroesophageal reflux disease

**DOI:** 10.1590/0102-672020260000026e1955

**Published:** 2026-08-21

**Authors:** Fernando Antonio Siqueira PINHEIRO, Jean Claude BOULEZ, Nelson Adami ANDREOLLO, Paulo KASSAB

**Affiliations:** 1 Universidade Federal do Ceará, Faculty of Medicine, Walter Cantídio University Hospital, Esophageal and Stomach Surgery Unit, Department of Surgery – Fortaleza (CE), Brazil.; 2 Claude Bernard University, Édouard Herriot Hospital, Digestive Surgery Service – Lyon, France.; 3 Universidade Estadual de Campinas, Department of Surgery – Campinas (SP), Brazil.; 4 Santa Casa de Misericórdia de São Paulo, Faculty of Medical Sciences, Department of Surgery, Gastroesophageal and Bariatric Surgery Unit – São Paulo (SP), Brazil.

**Keywords:** History of Medicine, Fundoplication, Gastroesophageal Reflux, Hernia, Hiatal, História da Medicina, Fundoplicatura, Refluxo Gastroesofágico, Hernia Hiatal

## Abstract

Rudolf Nissen published his first two cases in 1956, thus inaugurating the era of pathological gastroesophageal reflux surgery. The original fundoplication proposed by Nissen was initially associated with a gastropexy. Later, when the importance of hiatal hernia in the pathophysiology of the disease lost prominence, it began to be performed in isolation and was quickly adopted by other European and American schools, becoming the surgical treatment of choice for reflux esophagitis. From the 1970s onwards, with Nissen fundoplication increasingly used as the treatment of choice for gastroesophageal reflux and hiatal hernia, minor alterations were proposed to optimize results and reduce complications. Given this scenario, as we celebrate seven decades since the operation described by Rudolf Nissen, we are revisiting a principle rather than a technique: the permanent commitment to the critical evolution of surgical practice. Created as a result of his intuition and ingenuity, fundoplication is not static; it transforms, adapts, and improves in light of evidence and patient needs. Perhaps this is, ultimately, Nissen’s greatest legacy: not just an operation, but a way of thinking about surgery as a restless, rigorous, and profoundly patient-centered procedure. This is the perspective we must focus on as we move forward, honoring the past without losing sight of the future.

## ARTICLE HIGHLIGHTS

Rudolf Nissen published his first two cases in 1956, thus inaugurating the era of pathological gastroesophageal reflux surgery.

From the 1970s onwards, with Nissen fundoplication increasingly used as the treatment of choice for gastroesophageal reflux and hiatal hernia, minor alterations were proposed.

Nissen’s greatest legacy: not just an operation, but a way of thinking about surgery as a restless, rigorous, and profoundly patient-centered procedure.

## Summary

**CENTRAL MESSAGE:**

When Rudolf Nissen arrived in Switzerland to establish himself as a professor of surgery at the University of Basel in 1952, he already carried with him the idea that would eternally mark his name in the history of world digestive surgery. According to Mario Rossetti, who had the privilege of witnessing firsthand, as a resident, the first fundoplication performed by Nissen in 1955. In 1956, Nissen published his first two cases, thus inaugurating the era of surgical treatment of pathological gastroesophageal reflux. The original fundoplication proposed by Nissen was initially associated with a gastropexy. Later, when the importance of hiatal hernia lost prominence in the pathophysiology of the disease, it began to be performed in isolation, and was quickly adopted by other European and American schools, becoming the surgical treatment of choice in reflux esophagitis.

**PERSPECTIVES:**

When we celebrate seven decades since the operation described by Rudolf Nissen, we are revisiting a principle rather than a technique: the permanent commitment to the critical evolution of surgical practice. The fundoplication that was born from his intuition and ingenuity does not remain static; it transforms, adapts, and improves in light of evidence and patient needs. Rigorous indication, avoiding surgery on patients with predictive factors for failure, technical expertise in performing fundoplication and hiatoplasty, and adequate peri and postoperative care can be fundamental to the success of surgical treatment.Perhaps this is, ultimately, Nissen’s greatest legacy: not just an operation, but a way of thinking about surgery, restless, rigorous, and profoundly patientcentered. It is from this perspective that we must continue, honoring the past without losing sight of the future.

## RESUMO

Rudolf Nissen publicou seus dois primeiros casos em 1956, inaugurando assim a era do tratamento cirúrgico do refluxo gastroesofágico patológico. A fundoplicatura original proposta por Nissen foi inicialmente associada a uma gastropexia. Posteriormente, quando a importância da hérnia hiatal perdeu destaque na fisiopatologia da doença, passou a ser realizada isoladamente e foi rapidamente adotada por outras escolas europeias e americanas, tornando-se o tratamento cirúrgico de escolha na esofagite de refluxo. A partir da década de 1970, com a fundoplicatura de Nissen sendo cada vez mais utilizada como tratamento de escolha para refluxo gastroesofágico e hérnia hiatal, pequenas alterações foram propostas na tentativa de otimizar os resultados e reduzir as complicações. Diante desse cenário, ao celebrarmos sete décadas desde a operação descrita por Rudolf Nissen, revisitamos um princípio, e não uma técnica: o compromisso permanente com a evolução crítica da prática cirúrgica. A fundoplicatura que nasceu de sua intuição e engenhosidade não permanece estática; ela se transforma, se adapta e se aprimora à luz das evidências e das necessidades dos pacientes. Talvez esse seja, em última análise, o maior legado de Nissen: não apenas uma operação, mas uma maneira de pensar a cirurgia, inquieta, rigorosa e profundamente centrada no paciente. É a partir dessa perspectiva que devemos prosseguir, honrando o passado sem perder de vista o futuro.

## INTRODUCTION

 When Rudolf Nissen arrived in Switzerland to establish himself as a professor of surgery at the University of Basel in 1952, he already carried with him the idea that would forever secure his name in the history of world digestive surgery. Son of the famous German orthopedic surgeon Franz Nissen, Rudolf was born on September 9, 1896, in Neisse, Germany, now Nysa, in southwestern Poland. A brilliant young man, he found the perfect environment at home to become a doctor, and at the young age of 17, he entered the Faculty of Medicine at the University of Breslau/Munich, graduating in 1920. From 1921 to 1933, he was the favorite pupil and protégé of the great Professor Ferdinand Sauerbruch, one of the most important and influential surgeons of the 20th century. In 1927, accompanying Professor Sauerbruch, who had been invited to assume the chair of surgery at the University of Berlin, Nissen was hired as a professor of surgery at the same university, thus beginning his prolific teaching career. With Hitler’s rise to power in 1933, already married to fellow physician Ruth Becherer and enjoying great prestige in the European surgical community, Nissen decided to leave Germany due to antisemitism. Recommended by his mentor, he took up a position as professor of surgery in Istanbul, where he remained until 1939. After the start of World War II, Nissen decided to cross the Atlantic to become a Research Fellow at Harvard University, Boston. In the USA, he had a very active, happy, and productive life. Having obtained American citizenship in 1944, he held several positions, including head of the Department of Surgery at the Jewish Hospital and Director of Surgery at Maimonides Hospital, both hospitals in Brooklyn, New York. He was also a professor of surgery at Long Island College of Medicine, also in New York. In 1952, with the end of the war, after declining invitations to assume the chair of surgery in Hamburg and Vienna, he returned to Europe and settled as a professor at the University of Basel, Switzerland, where he would remain until his retirement in 1967^15^. 

### The idea

**Figure 1:**
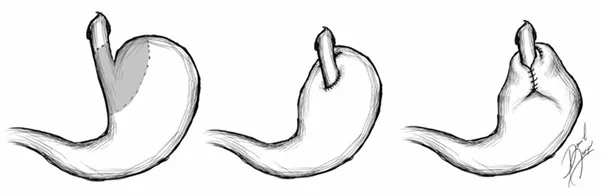
Transpleural resection of the cardia with tunnel esophagogastrostomy performed by Nissen in the 1930s Source: Personal archive.

 According to Mario Rossetti, who, as a resident, had the privilege of witnessing firsthand the first fundoplication performed by Nissen in 1955, his mentor was strongly influenced by the surprising quality of life his patients achieved after transpleural resections of the cardia with tunnel esophagogastrostomy in the 1930s, a continent anastomosis that protected the suture and prevented postoperative reflux^21,29,30^ (Figure 1). In 1956, Nissen published his first two cases, thus inaugurating the era of pathological gastroesophageal reflux surgery^22^. The original fundoplication proposed by Nissen was initially associated with a gastropexy^24^. Later, when the importance of hiatal hernia in the pathophysiology of the disease lost prominence, it began to be performed in isolation and was quickly adopted by other European and American schools, becoming the surgical treatment of choice for reflux esophagitis^21^. The initial enthusiasm for the operation gradually gave way to caution, as complications such as dysphagia, flatulence, and inability to belch and vomit soon appeared, leading to the emergence in the medical literature of the term “post-fundoplication syndrome,” which came to be used as a criticism of the procedure^15^. 

### Evaluation, evolution, and consolidation of the technique

 As with any new surgical technique, time was needed to properly evaluate results and complications. The symptoms of the so-described post-fundoplication syndrome were rarely observed in patients operated on by Professor Nissen^23^. That was not the case with some patients treated in other services, who ended up seeking treatment at the University Hospital of Basel for their complications. A retrospective analysis performed at the time raised the possibility of a technical error because some surgeons tended to perform a too-long and too-tight fundoplication. That triggered the emergence of experimental, clinical, and diagnostic research in the late 1960s, providing more information about the physiology of the esophagogastric junction. The important anatomical and physiological studies of Dorothea Liebermann-Meffert, daughter of Dr. Karl Peter Meffert, a friend of Nissen since his residency with Professor Sauerbruch, and who also settled in Switzerland, clearly revealed the architecture and functioning of the musculature of the esophagogastric tract^14-16^. Another important contribution was that of Nissen’s friend, Professor Jörg Rüdiger Siewert, from the University of Munich, who addressed topics such as the physiology, pathophysiology, and hormonal and pharmacological influences on the pressure of the lower esophageal sphincter, which were already being investigated at the time using esophageal manometry^32,33^. 

 Meanwhile, a true revolution took place in the fields of surgical and clinical treatment of gastroesophageal reflux disease (GERD). In 1963, at the French Academy of Surgery, André Toupet presented his 180º partial fundoplication after Heller’s cardiomyotomy^35^, which two years later was slightly modified by Lind et al. to reduce dysphagia and flatulence after total fundoplication^17^. Also in the late 1960s, Scotsman James Whyte Black was responsible for the research project at Smith, Kline & French that launched cimetidine on the market, an antagonist of histamine H2 receptors, as a strong suppressor of gastric acid secretion, drawing attention to its potential in the clinical treatment of peptic ulcer and reflux esophagitis. 

**Figure 2:**
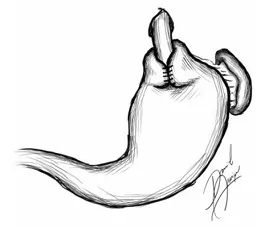
Anterior fundoplication without ligation of the short vessels, as proposed by Mario Rossetti^28,30^. Source: Personal archive.

 From the 1970s onwards, with Nissen fundoplication increasingly used as the treatment of choice for gastroesophageal reflux and hiatal hernia, minor alterations were proposed to optimize results and reduce complications. The first of these was the creation of an anterior fundoplication without ligation of the short vessels, proposed by Mario Rossetti in 1977 (Figure 2). With a case series of over 1,400 patients and head of the Department of Surgery at the University of Basel, the disciple and successor of Rudolf Nissen presented his data to the world and, despite the excellent results, did not immediately convince his peers^28,30^. 

**Figure 3:**
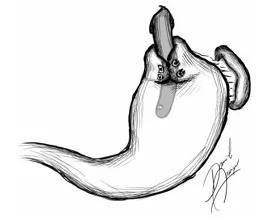
Fundoplication calibrated with a 60 French probe and a horizontal suture on a 2-0 polypropylene mattress, reinforced with Teflon pledgets, as proposed by Tom DeMeester^7^. Source: Personal archive.

 Around the same time, Donahue et al.^8^ launched the idea of creating a short, loose valve (floppy Nissen) and, in 1986, DeMeester et al.^7^, based on his clinical and laboratory studies to understand the physiology of the antireflux mechanism in humans, suggested a calibrated fundoplication with a 60 French probe, reduction of the valve length to 1.5–2.0 cm, performing the technique with a single U-shaped stitch using non-absorbable suture material, and ligating the short gastric vessels up to the upper third of the greater curvature to avoid lateral tension or torsion of the valve (Figure 3)^7^. 

 In the late 1980s, thirty years after the original idea, Nissen fundoplication was established as a safe and effective therapeutic option for GERD. Numerous studies have shown satisfactory short, medium, and long-term results, with low complication rates^4,7,8,10,31,34^. 

 In the early 1990s, with the positive impact of proton pump inhibitors^11^ in the clinical treatment of reflux esophagitis, the first Nissen fundoplication performed by videolaparoscopy in 1991 by Belgian surgeon Bernard Dallemagne was a watershed moment in the definitive popularization of Nissen fundoplication^6^. From then on, buoyed by the advantages of videolaparoscopy, an avalanche of articles emerged worldwide showing good results and important case series^5,12,26^. The initial technical difficulty of ligating the short vessels using minimally invasive surgery led some authors to revive the technique of anterior fundoplication without ligation of the short vessels proposed by Mario Rossetti, reproducing the excellent results demonstrated by him^9,20,27^. In Brazil, in the late 1990s, Lopes et al. published cases of patients who underwent a modified Nissen technique (mixed, total, and partial fundoplication) with good results^18^. 

### The legacy – between consensus and controversy

 Seven decades after its original proposal, Nissen fundoplication remains the world’s most frequently performed anti-reflux surgery, with thousands of procedures performed annually, despite the reduction caused by the overuse of proton pump inhibitors. Seventy years of accumulated experience have provided sufficient knowledge to fully understand how the procedure works and its short, medium, and long-term results and complications. GERD continues to be a very prevalent disease, a progressive condition that can lead to Barrett’s esophagus and esophageal adenocarcinoma. Therefore, surgical intervention is indicated in patients who require continuous medication and in those who are refractory to treatment with proton pump inhibitors, especially younger patients^2,13^. 

 Some issues remain controversial, such as surgical indication and outcomes in patients with atypical symptoms, patients with significant esophageal motility disorders, patients with large hiatal hernias and few symptoms, and obese patients^1,19,25,32^. Bonavina et al. draw attention to patient dissatisfaction with long-term clinical treatment, growing safety concerns associated with the prolonged use of proton pump inhibitors, and the increasing prevalence of GERD in the younger population. The authors propose an individualized surgical treatment for GERD, considering aspects such as adequate patient preparation, the correct technique for each patient, surgical team expertise, and even steps to more effectively promote positive surgical results^3^. In particular, we believe in and are working on creating routine strategies to maintain and even improve the positive results, especially in the long term. Rigorous indication, avoiding surgery on patients with predictive factors for failure, fundoplication and hiatoplasty technical expertise, and adequate peri and postoperative care can be fundamental to the success of surgical treatment. 

 Given this scenario, as we celebrate seven decades since the operation described by Rudolf Nissen, we are revisiting a principle rather than a technique: the permanent commitment to the critical evolution of surgical practice. Created as a result of his intuition and ingenuity, fundoplication is not static; it transforms, adapts, and improves in light of evidence and patient needs. Perhaps this is, ultimately, Nissen’s greatest legacy: not just an operation, but a way of thinking about surgery as a restless, rigorous, and profoundly patient-centered procedure. This is the perspective we must focus on as we move forward, honoring the past without losing sight of the future. 

## Data Availability

The datasets generated and/or analyzed during the current study are available from the corresponding author upon reasonable request.
